# Activity of the porcine gonadotropin-releasing hormone receptor gene promoter is partially conferred by a distal gonadotrope specific element (GSE) within an upstream enhancing region, two proximal GSEs and a retinoid X receptor binding site

**DOI:** 10.1186/s12958-015-0033-0

**Published:** 2015-05-17

**Authors:** Rebecca A Cederberg, Jacqueline E Smith, Emily A McDonald, Chanho Lee, Amy R Perkins, Brett R White

**Affiliations:** Laboratory of Reproductive Biology, Department of Animal Science, Institute of Agriculture and Natural Resources, University of Nebraska-Lincoln, Lincoln, Nebraska USA; Current address: Stowers Institute for Medical Research, Kansas City, MO USA; Current address: Center for International Health Research, Rhode Island Hospital, Providence, RI USA; Current address: Arizona Andrology Laboratory and Cryobank, Tuscon, AZ USA

**Keywords:** GnRH, GnRH receptor, Transcriptional regulation, Gene expression, Gonadotrope specific element, Steroidogenic factor 1, Retinoid X receptor, Anterior pituitary, Porcine

## Abstract

**Background:**

Regulation of gonadotropin-releasing hormone (GnRH) receptor (GnRHR) numbers on gonadotropes within the anterior pituitary gland represents a critical point for control of reproductive function. Binding of GnRH to its receptor regulates follicle stimulating hormone (FSH) and luteinizing hormone (LH) release and levels of this G-protein coupled receptor on the surface of gonadotropes determines their sensitivity to GnRH pulses. While transcriptional regulation of this gene has been studied in mice, rats, humans and sheep, little is known about its regulation in the pig, an important agricultural species and human research model.

**Methods:**

We isolated 5118 bp of 5′ flanking sequence for the porcine GnRHR gene and generated luciferase reporter vectors. Deletion and mutation constructs were evaluated in gonadotrope-derived alphaT3-1 cells to determine regions important for gene transcription. Additionally, electrophoretic mobility shift assays (EMSAs) were performed to identify transcription factors binding to the GnRHR promoter.

**Results:**

Transient transfections revealed that the GnRHR promoter was functional in alphaT3-1 cells but not in cells of non-gonadotrope origin. Mutation of the highly conserved gonadotrope specific element (GSE) located at -179/-171 of proximal promoter completely ablated luciferase activity, whereas mutation of another GSE at -315/-310 reduced activity by 34%. Consistent with this, EMSAs using alphaT3-1 nuclear extracts and a steroidogenic factor (SF)1 antibody confirmed SF1 binding to both GSEs. EMSAs also demonstrated that a retinoid X receptor (RXR) binding site at -279/-274 binds RXRalpha and RXRbeta and mutation of this site eliminated promoter activity. Transient transfection of alphaT3-1 cells with reporter vectors containing selective removal of 5′ flanking region for the porcine GnRHR gene indicated that the -1915/-1431 segment was important for promoter activity. Definition of this region via transfection assays and EMSAs revealed an upstream enhancing region located at -1779/-1667 that increases porcine GnRHR gene expression in alphaT3-1 cells and includes a SF1 binding site at -1760/-1753.

**Conclusions:**

Porcine GnRHR promoter activity in alphaT3-1 cells is partially conferred by a distal GSE, two proximal GSEs and a RXR binding site. Basal gonadotrope expression of the porcine GnRHR gene uniquely involves three GSEs and RXR is newly identified as a regulator of GnRHR promoter activity.

## Background

The interaction between gonadotropin-releasing hormone (GnRH) and its cognate receptor represents a central point for regulation of reproductive function. The pulsatile release of GnRH from the hypothalamus and its subsequent binding to specific, high-affinity receptors on gonadotrope cells of the anterior pituitary gland stimulates the up-regulation of genes encoding the common glycoprotein α-subunit and specific luteinizing hormone (LH)β- and follicle stimulating hormone (FSH)β-subunits that combine to produce LH and FSH [1-5]. In addition to changes in GnRH secretion, alterations in the number of pituitary GnRH receptors (GnRHR) have been implicated as an important regulatory mechanism of gonadotropin secretion [6]. Moreover, GnRH has been shown to regulate GnRHR mRNA levels in the anterior pituitary gland, indicating transcriptional regulation of the GnRHR gene as a method to regulate receptor levels on the plasma membrane of gonadotropes [7-10].

Basal expression of the mouse GnRHR gene in the gonadotrope-derived αT3-1 cell line is mediated by 600 bp of proximal promoter, comprised of a gonadotrope specific element (GSE), an activator protein (AP)-1 binding site and an element termed GnRH receptor activating sequence or GRAS [11-14]. In addition, basal activity of the mouse GnRHR promoter is dependent upon: LHX3, a member of the LIM homeodomain family [15]; two octamer transcription factor (OCT1) binding sites [16]; a pituitary homeobox (Pitx)-1 site that acts synergistically with an AP-1 element [17]; the sequence underlying responsiveness to GnRH (SURG)-1 that binds OCT1 and nuclear factor (NF)-Y [18]; and E-boxes that bind CLOCK protein [19]. Similar to the mouse, the rat GnRHR promoter contains AP-1, SF1 and GRAS elements in the proximal region [20]. Further, the rat GnRHR gene is regulated by the proximal promoter, the SF1 adjacent protein (SAP) and a distal region, termed GnRHR specific enhancer (GnSE) that interacts with GATA factors and the LIM-related factors, ISL1 and LHX3 [20-22]. Much less is known regarding basal expression of the human and sheep GnRHR genes. The human GnRHR promoter contains an SF1 binding site necessary for expression [23], an upstream AP-1 element (-1000/-994) involved in transcriptional down-regulation and an OCT1 binding site that acts as a repressor [24,25]. An SF1 binding site has been implicated in basal expression of the ovine GnRHR gene and 2700 bp of promoter confers tissue-specific expression in transgenic mice [26].

The porcine GnRHR gene is located on chromosome 8 and is near a quantitative trait locus for genes influencing ovulation rate [27-29]. A C/G substitution in the 3′ untranslated region (UTR) was shown to be significantly associated with numbers of corpora lutea [30]. Jiang and colleagues originally identified 1154 bp of proximal promoter for the porcine GnRHR promoter [30]. However, the elements necessary for transcriptional regulation of the porcine GnRHR gene remain to be elucidated. In this study, we isolated 5118 bp of 5′ flanking region for the porcine GnRHR gene and established its functionality in the gonadotrope-derived αT3-1 cell line. In addition, we identified two binding sites for SF1, a retinoid X receptor (RXR) binding site and a GSE in the upstream enhancing region that are necessary for basal expression of this gene.

## Methods

Experiments involving the use of recombinant DNA have been approved by the UNL Institutional Biosafety Committee under Protocol ID # 12 entitled: Functional Analysis of GnRHR I and II in Swine. The UNL Radiation Safety Office has approved the use of isotopes in the following experiments via AU License # I-387.

### Isolation of the porcine GnRHR gene promoter

Sequence for the porcine GnRHR promoter was obtained by inverse PCR [31] utilizing primers specific for 1154 bp of 5′ UTR previously reported by Jiang and colleagues (GenBank Accession No. AF227685) [30]. Briefly, genomic DNA preparations were partially cut by *Sau*3AI or *Eco*RI and fragments were self-ligated using a high concentration of T4 DNA ligase (4 U/μl, New England Biolabs, Beverly, MA) and a low concentration of DNA (3 ng/μl). Subsequently, circularized DNA fragments were amplified with the forward primer, 5′-TGG ACT GAC CGT TGA GAC TG-3′ and either the 5′-GTG TAA GTG TTG GAA CCA CAT C-3′ (*Sau*3AI digested DNA) or 5′-GAG AGC AAT AGC ATT CTC TG-3′ (*Eco*RI digested DNA) reverse primer to generate the inverse PCR products. The chosen primers were different from traditional PCR in that they were in reverse orientation. Resultant PCR products were subcloned and sequenced at the University of Nebraska-Lincoln (UNL) Genomics Core Research Facility. Sequence data for the 5171 bp of 5′ flanking region for the porcine GnRHR gene has been deposited with GenBank (Accession No. AY166667).

### Plasmids

A reporter vector containing 5118 bp of porcine GnRHR promoter (-5118pGL3) was constructed by PCR amplification of the 5′ flanking region for the GnRHR gene from genomic DNA preparations of pigs representing a white crossbred line (Table 1). The PCR product was subsequently subcloned into pBluescript SK- (Stratagene, La Jolla, CA). Promoter inserts were ligated into a reporter vector containing the cDNA encoding luciferase (pGL3, Promega Corp., Madison, WI) at the *SacI*/*EcoRV* location of the multiple cloning site. Deletion constructs were made by progressively removing 5′ flanking sequence (approximately 500 bp) via restriction enzyme digests (*ApaI*, *BstXI, NdeI, NsiI, BstAPI, PvuII, SpeI* and *BlpI*) and subsequent intramolecular ligation of the remaining vector backbone. Construction of the deletion constructs in the absence of restriction enzyme digest sites and 100-bp deletion reporter vectors were achieved by amplifying the specified region of the promoter by PCR (Table 1) with a high fidelity Taq DNA polymerase (Bioline, Springfield, NJ). Next, the fragments were subcloned into pBluescript SK- or pCR-Blunt II (Invitrogen, Carlsbad, CA), and then finally into pGL3. Block replacement vectors were constructed via an overlap extension, two-step, site-specific mutagenesis protocol [32] in which altered primers introduced the sequence changes (Table 1). The -5118pGL3 construct served as the template for the two first round PCR reactions where outer forward (OF) or outer reverse (OR) primers were used with the inner mutation containing primer or its complement (Table 1). The single second round PCR used the products from the first reactions as the template and OF and OR as the primer set (Table 1). The μSF1IpGL3, μSF1IIpGL3 and μSF1IIIpGL3 block replacement vectors were constructed by replacing the SF1 binding sites at -179/-171, -315/-310 and -1760/-1753 with *NotI*, *ClaI* and *NotI* sites respectively. The μRXRpGL3, μNF_Κ_BpGL3 and μOCT1pGL3 block replacement vectors were constructed by replacing the respective binding sites at -279/-274, -1689/-1684 and -1731/-1707 with *PstI*, *EcoRI* and *SacI*. To verify that the correct mutations had been introduced, vectors were sequenced at the UNL Genomics Core Research Facility before use in the transient transfection experiments. As a transfection efficiency control, luciferase vectors were co-transfected with a plasmid containing the Rous sarcoma virus (RSV) promoter linked to the cDNA-encoding β-galactosidase (RSV-β-gal, Stratagene). A midi plasmid preparation kit (Qiagen, Valencia, CA) was used to isolate transfection quality DNA.

**Table 1:** **Primers used to generate reporter vectors** ^**a**^

**Name**	**Sequence**
*-5118pGL3 F*	5′-CAGACAATTAGATTCCAGGGC-3′
*Promoter R*	5′-TCCTTCCCCAACTGATGTAG-3′
*-1810pGL3 F*	5′-GTTATGTGGAAGAGCCGGTG-3′
*-1716pGL3 F*	5′-TGGCTTGCAGAAACCTAACC-3′
*-1666pGL3 F*	5′-AGGCACTAATCCAGTGTCTGC-3′
*-1548pGL3 F*	5′-TACTCCTCTTGATTTCTGACTC-3′
*μSF1IpGL3 F*	5′-AAGTACACAAAACAAGTT*GCGGCCGC*TCTTTCACATTAAATATA-3′
*μSF1IIpGL3 F*	5′-ACAAAATTAAGCTTCGAA*ATCGAT*TCTTCACCTAGGAAAAAT-3′
*μRXRpGL3 F*	5′-AAAAATGTTGTGAAAACC*CTGCAG*TCTGCTGAGGTACTACAG-3′
*proximal OF*	5′-GTTATGTGGAAGAGCCGGTG-3′
*proximal OR*	5′-CTTTATGTTTTTGGCGTCTTCC-3′
*μSF1IIIpGL3 F*	5′-GAGTTTTGGTTTGTTTTA*GCGGCCGC*TTAGCAAATGAACCCTAT-3′
*μNF-κBpGL3 F*	5′-GAAACCTAACCCCATATT*GAATTC*GAGAGCAATGGTTCAGTA-3′
*μOCT1pGL3 F*	5′-CAAATGAACCCTATGTGA*GAGCTC*TGGTGTTTTGGCTTGCAG-3′
*distal OF*	5′-CAGAGAATGCTATTGCTCTC-3′
*distal OR*	5′-GTGTAAGTGTTGGAACCACATC-3′

^a^Block replacement mutation reporter vectors were generated with outer forward (OF) or outer reverse (OR) primers as described in the Methods. The proximal OR resides in pGL3 3′ of the insert. Italicized bases indicate the new restriction enzyme digest site that replaced the transcription factor binding site in the native sequence.

### Cell culture and transfections

Cultures of αT3-1 (mouse gonadotrope, Dr. Pam Mellon, Salk Institute, La Jolla, CA), CHO (Chinese hamster ovary), Cos-7 (monkey kidney), JAR (human choriocarcinoma), MA10 (mouse Leydig) and PK15 (pig kidney) cells were maintained at 37°C in a humidified 5% CO_2_ in air atmosphere. The αT3-1 cells were cultured in high-glucose (4.5 g/L) DMEM (Mediatech, Herndon, VA) supplemented with 5% fetal bovine serum (FBS), 5% horse serum, 2 mM glutamine, 100 U/ml penicillin, and 100 μg/ml streptomycin sulfate (Gibco, Grand Island, NY). The CHO, Cos-7 and PK15 cell lines were cultured in high-glucose (4.5 g/L) DMEM supplemented with 10% FBS, 2 mM glutamine, 100 U/ml penicillin, and 100 μg/ml streptomycin sulfate. Finally, JAR and MA10 cells were cultured in Roswell Park Memorial Institute-1640 medium (RPMI-1640; Mediatech) supplemented with 10% FBS, 100 U/ml penicillin, and 100 μg/ml streptomycin sulfate.

Transient transfections were performed using a liposome-mediated protocol (Fugene6; Roche Diagnostics Corp., Indianapolis, IN) following manufacturer’s instructions. The day prior to transfection, cells were plated in 6-well tissue culture plates at the appropriate density (between 2 × 10^5^ and 2 × 10^6^ cells) to result in 40% confluency on the day of transfection. Cells were transfected with a 3:1 Fugene6 to DNA ratio. A total of 1 μg of DNA, 0.9 μg of luciferase test vector and 0.1 μg of RSV-βgal were used per well. Luciferase (Promega Corp.) and β-galactosidase assays (β-gal; Applied Biosystems, Bedford, MA) were performed following manufacturer’s instructions. At 20-24 h post transfection, cells were washed twice with ice-cold PBS and harvested with 200 μl of lysis buffer [100 mM potassium phosphate (pH 7.8), 0.2% Triton X-100 and 1 mM dithiothreitol (DTT)]. Lysates were cleared by centrifugation (14,000 × *g*) for 2 min at 4°C and 20 μl of lysate was used to perform each of the assays. Luciferase and β-gal values for each sample were determined using a Wallac VICTOR^2^ micro plate reader (PerkinElmer Life Sciences, Boston, MA). To normalize for transfection efficiency, luciferase activity was divided by β-gal values.

### EMSA

Nuclear protein extracts were prepared from αT3-1 cells utilizing the NE-PER® Nuclear and Cytoplasmic Extraction Reagent Kit per manufacturer’s instructions (Pierce Biotechnology, Rockford, IL). Approximately 2.8 × 10^8^ αT3-1 cells were harvested with TNE buffer [10 mM Tris (pH 8), 140 mM NaCl, 1 mM EDTA] and the extracts were treated with protease (catalog no. P8340, Sigma Chemical Co., St. Louis, MO) and phosphatase (catalog no. 524625, Calbiochem, La Jolla, CA) inhibitor cocktails to prevent enzymatic degradation of proteins. The amount of protein present in the extracts was determined using bicinchoninic acid (BCA assay; Pierce Biotechnology). Double-stranded oligonucleotide probes (Table 2) were end-labeled with [γ-^32^P]ATP using T4 polynucleotide kinase (MBI Fermentas Inc., Hanover, MD) and purified using sephadex G-25 spin columns (Amersham Biosciences Corp., Piscataway, NJ). Nuclear extracts (5 μg) were incubated with 4 μl of Dignam D buffer (20 mM HEPES, 20% glycerol, 0.1 M potassium chloride, 0.2 mM EDTA, 0.5 mM DTT), 1 mM DTT, 2 μg poly(dI•dC) (Amersham Biosciences Corp.) and, where indicated, rabbit antiserum directed against Ad4BP/SF1 (including the DNA binding domain; Dr. Ken-ichirou Morohashi, Okazaki National Research Institutes, Okazaki, Japan), mouse monoclonal antibodies for RXRα, RXRβ and RXRγ (Dr. Pierre Chambon, Institut de Génétique et de Biologie Moléculaire et Cellulaire, Illkirch Cedex, France) or an equal amount of rabbit IgG (catalog no. sc-2027, Santa Cruz Biotechnology, Santa Cruz, CA) at 25°C for 30 min. Following incubation, radiolabeled probe (100,000 cpm) was added and, where indicated, 50-fold molar excess of either homologous, heterologous, consensus or mutant consensus unlabeled competitor. Reactions were incubated for an additional 20 min at 25°C before free probe was separated from bound probe by electrophoresis for 1.5 h at 30 mA in 5% polyacrylamide gels that were pre-run for 1 h at 100 V in 1 X TGE [25 mM Tris (pH 8.3), 190 mM glycine and 1 mM EDTA]. Gels were transferred to blotting paper, dried, and exposed to Biomax MS film (Eastman Kodak Co. Rochester, NY) or RX-B Blue Sensitive autoradiographic film (Marsh BioProducts, Rochester, NY) for 20-24 h at -80°C before being developed.

**Table 2:** **Sense strand of EMSA oligonucleotides** ^**a**^

**Name**	**Sequence**
*-184/-165 homologous*	5′-CAAGTTCACCTTGATCTTTC-3′
*-184/-165 heterologous*	5′-CAAGTCTACCGTATTCTTT-3′
*-323/-303 homologous*	5′-GCTTCGAATGTCCTTCTTCAC-3′
*-323/-303 heterologous*	5′-CTGCAGCAACATAACTACTAC-3′
*-290/-270 homologous*	5′-TTGTGAAAACCAGGCCATCTG-3′
*-290/-270 heterologous*	5′-CTAGGAAAAATGTTGTGAAAA-3′
*-1810/-1780 homologous*	5′-GTTATGTGGAAGAGCCGGTGTTCAAAACTGA-3′
*-1779/-1749 homologous*	5′-TGAGTTTTGGTTTGTTTTACAAGGACATTAG-3′
*-1748/-1717 homologous*	5′-CAAATGAACCCTATGTGATGCAAATGGTGTTT-3′
*-1737/-1707 homologous*	5′-TATGTGATGCAAATGGTGTTTTGGCTTGCAG-3′
*-1716/-1691 homologous*	5′-TGGCTTGCAGAAACCTAACCCCATAT-3′
*-1690/-1667 homologous*	5′-TTCCACTGAGAGCAATGGTTCAGT-3′
*-1810/-1667 heterologous*	5′-CTGCAGCAACATAACTACTAC-3′
*SF1 consensus*	5′-CCAGTTCACCTTGATCTTTC-3′
*RXR consensus*	5′-AGCTTCAGGTCAGAGGTCAGAGAGCT-3′
*NF-Y consensus*	5′-AGACCGTACGTGATTGGTTAATCTCTT-3′
*OCT1 consensus*	5′-TGTCGAATGCAAATCACTAGAA-3′
*μOCT1 consensus*	5′-TGTCGAATGCAAGCCACTAGAA-3′

^a^The complement strand was annealed for each oligonucleotide prior to use in EMSAs.

### Bioinformatics and statistical analysis

Initial sequence compilation was performed with the GCG Wisconsin Package (Accelrys Software Inc., San Diego, CA). Additional sequence management and alignment procedures were completed with Vector NTI software (InforMax, Bethesda, MD). Species comparisons were primarily performed utilizing *vis*ualization *t*ool for *a*lignment (VISTA) [33] and sequences were aligned with Vector NTI software. Analyses of sequence for transcription factor binding sites were performed with the Patch Public 1.0 program (Biobase, Wolfenbüttel, Germany).

Data were analyzed using the general linear models (GLM) procedure of the Statistical Analysis System software (version 8.2, SAS Institute Inc, Cary, NC). To control for transfection efficiency, the arbitrary light value for each luciferase replicate was divided by the respective β-gal arbitrary light value and this value was then divided by the mean of the empty vector and expressed as fold over pGL3. Transfections were performed in triplicate with three to five replicates containing different plasmid preparations. Individual values (n = 9-15) from all the replicates were used to generate the mean ± SEM. Comparisons between pGL3 and test vectors were analyzed using Dunnett’s t-test. Means for luciferase activity among test vectors were compared using the least significant differences of least squares means.

## Results

### The porcine GnRHR promoter has limited homology to other species

We isolated the porcine GnRHR promoter (GenBank Accession No. AY166667) and determined sequence similarity among species to assist in identification of regulatory elements. Direct sequence alignment of 682 bp of proximal GnRHR promoter indicated that the pig had greater homology to the cow (78.7%), sheep (76.6%) and human (72.3%) than to the mouse (61.8%) or rat (61.6%). When VISTA [33] was used to examine species similarities, we identified 2 to 3 regions per species with at least 75% identity of over 100 bp or more. Compared to the pig, GnRHR promoters from the human, cow and sheep have a 400-bp region of homology immediately upstream of the translational start site, whereas the mouse and rat promoters only have about 250 bp of similar sequence. In this region of promoter, every species has a functional or putative GSE (Table 3). Interestingly, the sheep, cow and proximal pig SF1 binding sites are positioned at essentially the same location, whereas the SF1 binding sites in human and rodents are located within 75 bp upstream or downstream of the proximal porcine GSE (-179/-171). Although the amino acid sequence for the GnRHR protein is more than 85% conserved among mammalian species, we determined that promoters for the GnRHR gene in mammals have minimal sequence homology beyond 400 bp upstream of the translational start site, suggesting that regulation of this gene is less conserved than its protein and function [34]. Additionally, DNA analysis revealed that transcription factor binding sites often had only 75-85% homology to those documented in other species, indicating that search parameters needed to be reduced to identify potential sites.

**Table 3:** **Comparison of SF1 binding sites identified in gonadotrope-specific genes**

**Gene**	**SF1 site**	**Location**
*Porcine GnRHR*	TCACCTTGA	-179/-171
*Porcine GnRHR*	TGTCCT	-315/-310
*Porcine GnRHR*	CAAGGACA	-1760/-1753
*Ovine GnRHR* [45]	TCACCTTGA	-178/-170
*Murine GnRHR* [14]	TGGCCTTCA	-244/-236
*Rat GnRHR* [20]	TGGCCTTCA	-245/-237
*Human GnRHR* [23]	CAGGGACA	-143/-136
*Bovine GnRHR* ^*a*^	TCACCTTGA	-178/-170
*Equine LHβ* [36]	TGACCTTG	-119/-106
*Equine LHβ* [36]	TGGCCTTG	-55/-48
*Rat LHβ* [46]	TGACCTTGT	-127/-119
*Rat LHβ* [46]	TGGCCTTGC	-59/-51

^a^Indicates a putative binding site.

### The porcine GnRHR promoter is functional in gonadotrope-derived αT3-1 cells, but not in cell lines of non-gonadotrope origin

**Figure 1:**
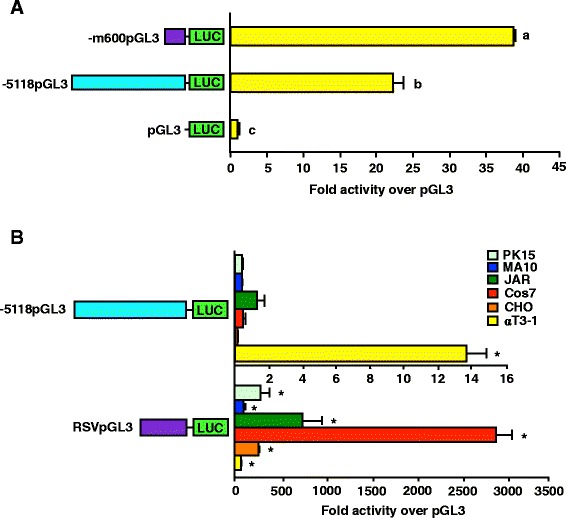
The -5118 porcine GnRHR promoter is cell-specific and functional in gonadotrope-derived αT3-1 cells. **A**, Cells were transfected with -5118pGL3, -m600pGL3 or pGL3. *Unique letters* indicate values that are significantly different from one another (*P* < 0.05). **B**, Transfections were performed with cell lines derived from gonadotrope or non-gonadotrope origin. Cells were transfected with -5118pGL3 (*upper panel*) or RSVpGL3 (*lower panel*) and expressed as activity over pGL3. An *asterisk* indicates values, within each test vector, that are greater than pGL3 (*P* < 0.05).

The αT3-1 cell line was transiently transfected with luciferase reporter vectors containing either the full-length porcine GnRHR promoter (-5118pGL3), -600 bp of GnRHR promoter from the mouse (-m600pGL3), previously shown to have robust activity in this cell line [11], or promoterless control (pGL3). Although luciferase activity was significantly reduced compared to that of the -m600pGL3 construct (approximately 50%), cells transfected with the porcine promoterconstruct (-5118pGL3) had luciferase levels that were more than 20-fold higher than promoterless controls (*P* < 0.05; Figure 1A). This result was interesting, especially given the fact that the human GnRHR promoter only exhibits 4-fold increased activity over promoterless control [23] and the ovine GnRHR promoter is inactive [26] in αT3-1 cells. Therefore, the αT3-1 cell line was identified as a model system to examine transcriptional regulation of the porcine GnRHR gene.

Cell lines from gonadotrope and non-gonadotrope origins were transiently transfected with the -5118pGL3 vector or a luciferase reporter vector containing a constitutively active promoter (RSVpGL3) as a positive control. The cell types transfected included: αT3-1, CHO, Cos-7, JAR, MA10 and PK15. Luciferase activity of the -5118pGL3 construct was greater than promoterless controls (*P* < 0.05) in only the gonadotrope-derived αT3-1 cell line (Figure 1B). The lack of promoter activity in cells of non-gonadotrope derivation was not due to our transfection method as cells transfected with RSVpGL3 had significantly higher levels of luciferase activity than promoterless controls (*P* < 0.05) in all cell types. Thus, the porcine GnRHR promoter was active in gonadotrope-derived cells, but not cell lines derived from ovary, kidney, placenta or testis tissues.

### Two proximal SF1 binding sites located at -179/-171 and -315/-310 are important to activity of the porcine GnRHR promoter in αT3-1 cells

**Figure 2:**
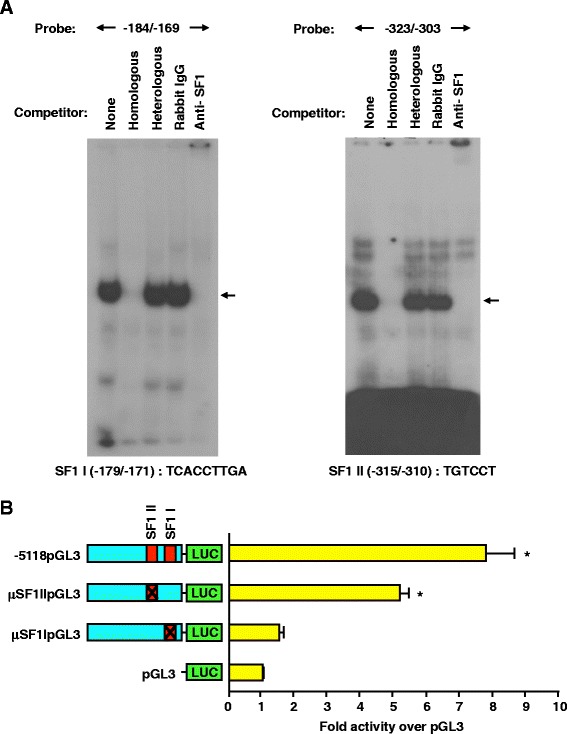
Proximal GSEs located at -179/-171 and -315/-310 of the porcine GnRHR promoter bind SF1. **A**, EMSAs performed with nuclear extracts and probes for the regions spanning either SF1 I or SF1 II. In addition, nuclear extracts were incubated with antiserum directed against SF1 (including its DNA binding domain) or normal rabbit IgG before the addition of radiolabeled probe (specific complex abrogation indicated by arrow). Electrophoresis of the SF1 I EMSA (left) was performed for an extended time, thus, free probe was run off the gel. **B**, Cells were transfected with -5118pGL3, μSF1IpGL3, μSF1IIpGL3 or pGL3. An *asterisk* indicates values that are greater than pGL3 (*P* < 0.05).

Given the conserved presence of GSEs in previously reported GnRHR promoters from other species, we investigated the role of SF1 in gonadotrope-specific expression of the porcine GnRHR gene. Sequence analysis of the proximal porcine GnRHR promoter identified two putative SF1 binding sites located between -179/-171 and -315/-310 relative to the translational start site (Table 3). To confirm SF1 binding to the GSEs identified within the porcine GnRHR promoter, EMSAs were performed with αT3-1 nuclear extracts and radiolabeled probes generated from the native promoter sequence for each of the SF1 binding sites at -179/-171 and -315/-310 (Table 2). Results indicated binding complexes for both oligonucleotide probes (Figure 2A). Specificity of DNA:protein interactions were assessed by competition with 50-fold molar excess of homologous or heterologous unlabeled DNA (specific complex indicated by arrow). Furthermore, binding complexes for both SF1 probes were abrogated with the addition of an antiserum against SF1 (including the DNA binding domain), whereas rabbit IgG had no effect (Figure 2A). Therefore, both the proximal (-179/-171) and distal (-315/-310) GSEs within the 5′ flanking region for the porcine GnRHR promoter bind SF1 from αT3-1 cell nuclear extracts.

To evaluate the function of the two GSEs, we transiently transfected αT3-1 cells with luciferase reporter constructs containing block replacement mutations of either the proximal (μSF1IpGL3) or distal (μSF1IIpGL3) SF1 binding sites, within the context of the -5118 promoter, and the wild-type full-length porcine promoter (-5118pGL3). Mutation of the distal GSE located at -315/-310 (μSF1IIpGL3) caused a 34% reduction in luciferase activity (*P* < 0.05) when compared to the full-length promoter (Figure 2B). More importantly, mutation of the proximal SF1 binding site (μSF1IpGL3), which has 100% homology to the GSE identified in the ovine GnRHR promoter, resulted in complete ablation of promoter activity (Figure 2B). Thus, the proximal SF1 binding site is necessary for gonadotrope expression of the porcine GnRHR gene, whereas the distal GSE is important for maximal basal expression.

### RXRα and RXRβ bind the porcine GnRHR promoter and mutation of the RXR binding site at -279/-274 abolishes activity of the promoter in αT3-1 cells

**Figure 3:**
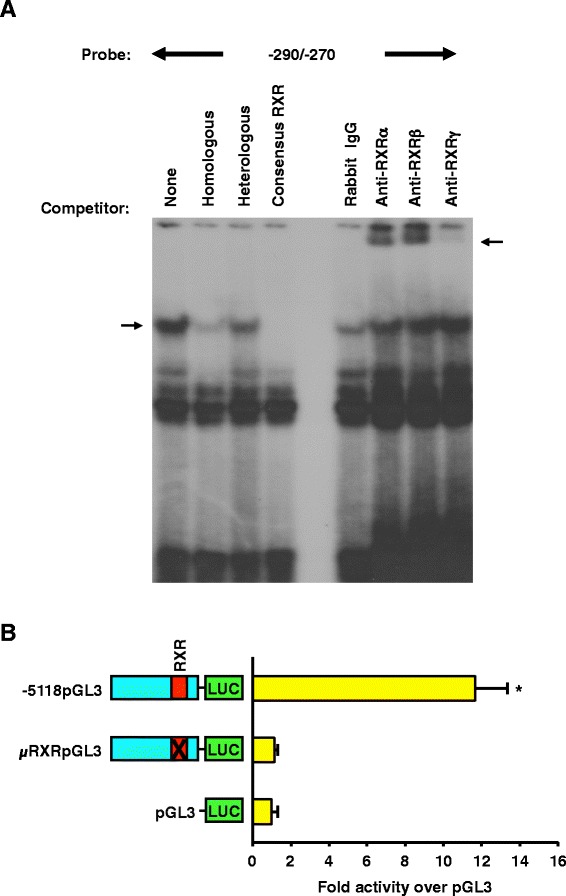
The RXR binding site important for GnRHR promoter activity (-279/-274) binds RXRα and β. **A**, EMSAs performed with nuclear extracts and a probe for the RXR region of the promoter (specific complex indicated by arrow). Nuclear extracts were also incubated with antibodies directed against the RXR subtypes (α, β and γ) or rabbit IgG before the addition of probe (supershift indicated by arrow). **B**, Cells were transiently transfected with -5118pGL3, μRXRpGL3 or pGL3. An *asterisk* indicates values that are greater than pGL3 (*P* < 0.05).

Transcription factor searches of the proximal GnRHR promoter identified a dense region of potentially relevant transcription factor binding sites between -290 and -270 bp of 5′ flanking sequence. EMSAs with a probe for the -290/-270 bp region relative to the translational start site resulted in a specific protein-DNA complex that was competed by homologous and consensus RXR probes, but unaffected by a heterologous oligonucleotide (Figure 3A). Addition of monoclonal antibodies specific for RXR subtypes (α, β and γ) resulted in super shifts for RXRα and RXRβ. To evaluate the functionality of the RXR complex, a luciferase reporter vector containing a block replacement of the RXR binding site at -279/-274 within the context of the -5118 GnRHR promoter (μRXRpGL3) was constructed. αT3-1 cells were transiently transfected with either μRXRpGL3, -5118pGL3 or pGL3. Mutation of the RXR site resulted in a significant (*P* < 0.05) and complete reduction of luciferase activity when compared to the full-length promoter (Figure 3B). Therefore, the RXR binding site at -279/-274 is essential for activity of the porcine GnRHR promoter in αT3-1 cells and the proteins RXRα and RXRβ bind this location of the promoter.

### Porcine GnRHR promoter activity in a gonadotrope-derived cell line is partially regulated by factors within the -1915/-1431 region

**Figure 4:**
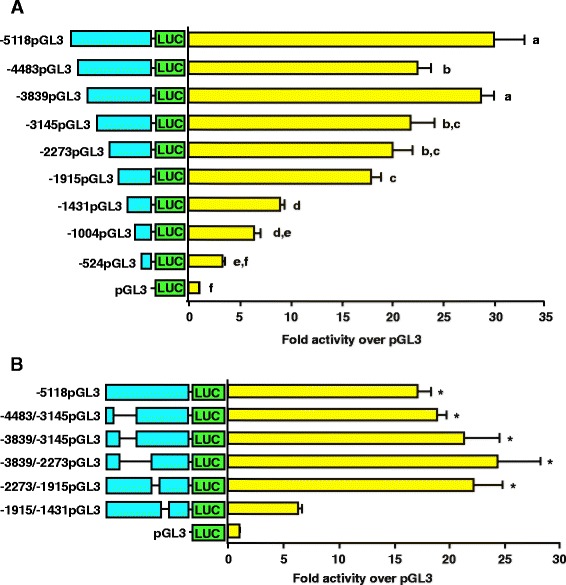
The -1915/-1431 bp distal portion of the GnRHR gene is important for gonadotrope expression. **A**, Cells were transfected with -5118pGL3 or pGL3. *Unique letters* indicate values that are significantly different from one another (*P* < 0.05). **B**, Transfections of cells with vectors harboring deleted blocks of internal sequence within -5118pGL3 or pGL3. An *asterisk* indicates values that are greater than pGL3 (*P* < 0.05).

Luciferase reporter vectors containing truncations of 5′ flanking sequence for the porcine GnRHR gene were transiently transfected into αT3-1 cells. Although some statistically significant differences were detected among deletion vectors within -5118/-1915 (*P* < 0.05), functionally, little luciferase activity was lost until the proximal promoter was reduced from -1915 to -1431. Consistent with this, activity of the -1431pGL3 construct was approximately 50% that of -1915pGL3. Additional losses in luciferase activity were identified (*P* < 0.05) as the promoter was further diminished from -1431 to -524, which was not significantly different from promoterless controls (Figure 4A). To eliminate the 5′ flanking region located between -5118/-1915 of the porcine GnRHR gene as a potential basal enhancer, we performed transient transfections in αT3-1 cells with reporter plasmids containing internally deleted blocks of sequence within this DNA fragment (Figure 4B). Only one of the reporter constructs decreased luciferase activity compared to the full-length -5118pGL3 vector (*P* < 0.05). Selective removal of the -1915/-1431 region reduced promoter activity by approximately 60%, confirming that this 484-bp sequence contains one or more important elements required for maximal basal activity of the porcine GnRHR gene promoter in the αT3-1 cell line (Figure 4B).

### Important regulatory elements are located within an upstream enhancing region at -1779/-1667 of the porcine GnRHR promoter

**Figure 5:**
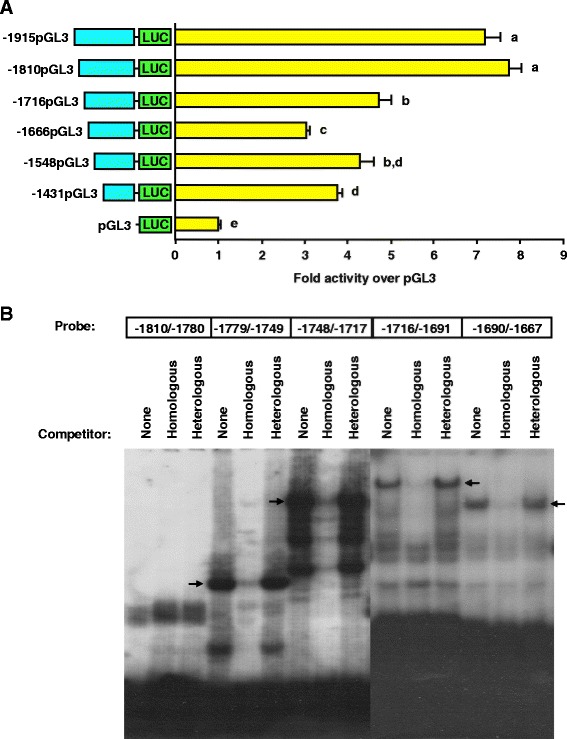
Activity of the porcine GnRHR promoter is dependent on an important upstream enhancing region. **A**, Cells were transfected with vectors containing sequential removal of 5′ flanking sequence in -1915pGL3 or pGL3. *Unique letters* indicate values that are significantly different from one another (*P* < 0.05). **B**, EMSAs performed with nuclear extracts and probes spanning the -1810/-1667 region of the porcine GnRHR promoter. Specificity of DNA-protein interactions was assessed by competition with excess homologous or heterologous DNA (arrow indicates specific complexes).

To further define the -1915/-1431 promoter region, we generated reporter constructs containing sequential 50- to 100-bp deletions of this sequence within the context of the -1915 porcine GnRHR promoter. Transient transfection of luciferase reporter vectors containing -1915, -1810, -1716, -1666, -1548 or -1431 bp of proximal promoter into αT3-1 cells resulted in a 46% decrease in luciferase activity (*P* < 0.05) upon reduction of the promoter from -1810 to -1716 bp (Figure 5A). An additional 36% loss in activity (*P* < 0.05) was observed when the -1716 GnRHR promoter was further diminished to -1666 bp, whereas reduction of the promoter to -1548 or -1431 bp did not decrease luciferase activity (Figure 5A). Therefore, transfection experiments indicated that the -1810/-1666 region contained distal elements that partially confer cell-specific activity of the porcine GnRHR promoter.

To refine the boundaries of this emerging distal enhancing region, EMSAs were performed with nuclear extracts from αT3-1 cells and five radiolabeled oligonucleotide probes spanning the -1810/-1667 region of the porcine GnRHR promoter. Results indicated specific binding complexes for four of the DNA probes, corresponding to the -1779/-1667 region upstream of the translational start site (Figure 5B). Sequence analysis of this region identified several putative transcription factor binding sites including: CCAAT enhancer binding protein (C/EBP), nuclear factor (NF)-κB, NF-Y, SF1 and the POU-domain DNA binding factor, OCT1. Thus, our laboratory has isolated a novel 113-bp region containing several putative elements that are essential to transcriptional regulation of the porcine GnRHR gene. Alignments of this region with genomic sequence databases from the mouse, rat and human indicated that there is not a comparable conserved sequence in the distal promoters of these species. However, regions of nearly 80% homology to these 113-bp were found in ovine, equine and bovine genome databases located at -2045/-1932, -1823/-1713 and -2288/-2176 relative the translational start site, respectively.

### Binding of SF1 to a recognition site located at -1760/-1753 bp of proximal promoter represents the first factor of an upstream enhancing region

**Figure 6:**
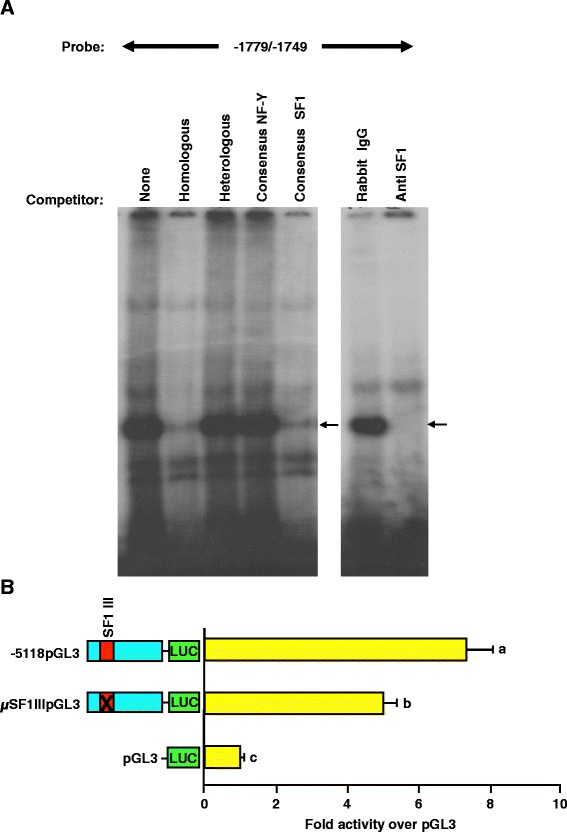
A third GSE within an upstream enhancing region is important for maximal basal activity of the GnRHR promoter. **A**, EMSAs were performed with nuclear extracts and oligonucleotides spanning -1779/-1749 bp of promoter. Along with homologous and heterologous, unlabeled oligonucleotides containing consensus binding sequences for NF-Y or SF1 were added (arrow indicates specific complex). An antibody directed against SF1 (including its DNA binding domain) or an equal mass of rabbit IgG were included (arrow indicates abrogation of complex). **B**, Transfections of cells with -5118pGL3, μSF1IIIpGL3 or pGL3. *Unique letters* indicate values that are significantly different from one another (*P* < 0.05).

To evaluate the relevance of the putative GSE located within this region, EMSAs were performed. A radiolabeled oligonucleotide spanning the SF1 binding site located at -1760/-1753 bp of 5′ flanking region for the porcine GnRHR gene was incubated with αT3-1 nuclear extracts. The addition of either homologous or heterologous DNA identified a specific binding complex (Figure 6A). Inclusion of unlabeled oligonucleotides containing consensus binding sites for NF-Y or SF1 resulted in competition by the consensus SF1 oligonucleotide (Figure 6A). Addition of an SF1 antiserum (including the DNA binding domain) ablated the DNA:protein complex, whereas rabbit IgG had no effect (Figure 6A). Thus, SF1 interacts with the GSE at -1760/-1753, which is located in a potentially important distal enhancer of the GnRHR promoter.

Reporter assays demonstrated that this SF1 binding site is functionally significant to the porcine GnRHR promoter. Luciferase reporter vectors containing the native full-length promoter (-5118pGL3), a block replacement mutation of the SF1 binding site, within the context of the -5118 promoter (μSF1IIIpGL3) or pGL3 were transiently transfected into αT3-1 cells. Cells transfected with μSF1IIIpGL3 had approximately a 30% reduction in luciferase activity (*P* < 0.05) compared to cells transfected with the full-length promoter (Figure 6B). Therefore, this SF1 binding site represents an additional distal member of the cell-specific promoter for the porcine GnRHR gene.

### Putative OCT1 and NF-κB elements identified within the upstream enhancing region do not contribute to functional activity of the porcine GnRHR gene promoter in αT3-1 cells

**Figure 7:**
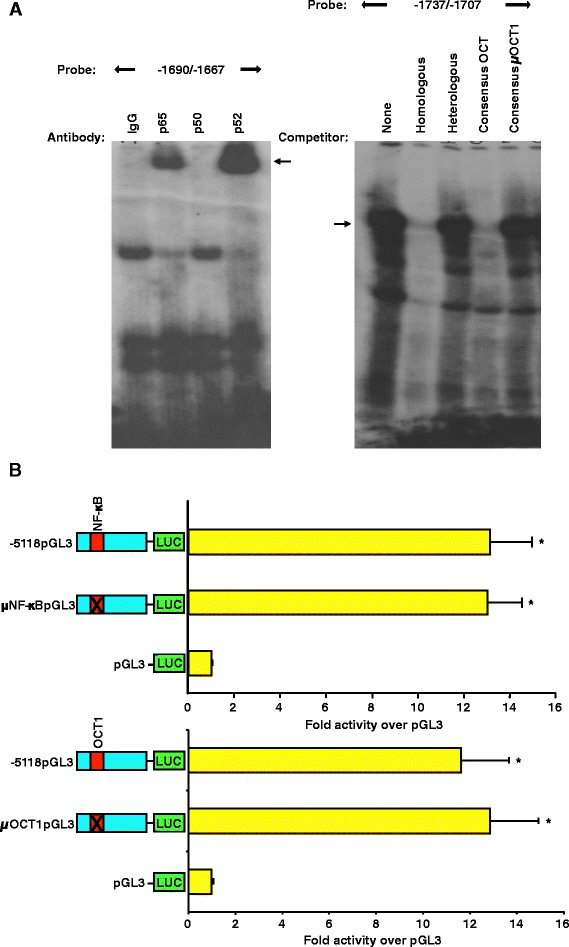
The putative NF-κB and OCT1 binding sites in the upstream enhancing region are not required for promoter activity. **A**, EMSAs were performed with nuclear extracts and probes for the -1690/-1667 region of the promoter, resulting in specific complexes. Antibodies for p65, p50 and p52 subunits of NF-κB or rabbit IgG were incubated with the probe (arrow indicates supershift). Nuclear extracts were incubated with the -1737/-1707 probe and specificity of the complex was tested with unlabeled homologous, heterologous, consensus OCT and mutated consensus OCT oligonucleotides (arrow indicates specific complex). **B**, Cells were transfected with -5118pGL3, μNF-κBpGL3 or pGL3. In another experiment, cells were transfected with -5118pGL3, μOCT1pGL3 or pGL3. An *asterisk* indicates values that are greater than pGL3 (*P* < 0.05).

Evaluation of the putative OCT1 and NF-κB binding sites identified within the enhancing region was performed via EMSAs. Radiolabeled oligonucleotides spanning the NF-κB binding site located at -1689/-1684 bp of proximal promoter incubated with αT3-1 nuclear extracts formed a specific binding complex that was competed by the addition of unlabeled homologous but not heterologous DNA (data not shown). Inclusion of antibodies specific for the p65, p50 and p52 subunits of NF-κB resulted in a supershift of the p65 and p52 subunits of NF-κB (Figure 7A). In addition, EMSAs using αT3-1 nuclear extracts and a radiolabeled oligonucleotide spanning the OCT1 element located at -1731/-1724 bp of 5′ flanking region revealed a binding complex. Specificity was indicated by the competition of complexes with the addition of unlabeled homologous and consensus OCT1 probes, but not heterologous and mutated consensus OCT1 oligonucleotides (Figure 7A). However, αT3-1 cells transiently transfected with luciferase reporter vectors containing block replacement mutations for either the NF-κB (μNF-κBpGL3) or OCT1 (μOCT1pGL3) elements did not differ in luciferase activity compared to the full-length porcine GnRHR promoter (-5118pGL3; Figure 7B). Thus, despite recruitment of binding factors in αT3-1 cells, the OCT1 and NF-κB elements were not functionally relevant to cell-specific expression of the porcine GnRHR gene.

## Discussion

Use of an *in vitro* cell culture system to evaluate functionality of the porcine GnRHR promoter allowed us to efficiently elucidate important elements required for basal expression of this gene in gonadotrope cells of the anterior pituitary gland. To this end, we identified three SF1 binding sites. Involvement of SF1 in transcriptional regulation of the GnRHR gene has been previously shown in several other species, including the human [23], mouse [14], rat [20] and sheep [26]. Mutation of the most proximal porcine GSE (-179/-171) resulted in complete ablation of promoter activity. In contrast, mutation of the two distal GSEs (-315/-310 and -1760/-1753) reduced activity of the promoter by 34% and 30% respectively, suggesting an important, albeit lesser role for these two SF1 binding sites. In contrast, activity of the mouse GnRHR promoter in αT3-1 cells was reduced by 60% when the SF1 binding site was mutated [14]. Human GnRHR promoter activity in αT3-1 cells was increased by 2.5-fold upon over-expression of SF1 and decreased by approximately 50% after addition of similar quantities of an SF1 antisense vector [23].

Additionally, SF1 is important for the expression of other gonadotropic genes, including the common glycoprotein α-subunit [35] and specific LHβ- [36,37] and FSHβ-subunits [38]. While expression of the LHβ-subunit gene in several species requires two GSEs [36], results described within this manuscript have implicated the porcine promoter as the only GnRHR promoter with three functional SF1 binding sites required for basal activity (Table 3). In contrast to the dual SF1 binding sites in the equine and rat LHβ-subunit promoters, the sequences for the two porcine proximal GSEs (-179/-171 and -315/-310) are quite different. Studies examining regulation of the murine FSHβ-subunit gene promoter in LβT2 cells revealed that mutation of either proximal GSE sequence did not affect promoter activity except when a downstream NF-Y site was also mutated; alteration of both sites decreased luciferase activity by 50% [38]. However, contrary to the study of other GnRHRs, we have identified three SF1 binding sites that are involved in the transcriptional regulation of the porcine GnRHR. The human steroidogenic acute regulatory protein (StAR) also contains three GSEs necessary for maximal promoter activity that are located in a similar arrangement (-42/-35, -105/-95 and -926/-918) as the GSEs within the swine GnRHR promoter. [39]. In addition to a SF1 binding site, cell-specific expression of the mouse and rat GnRHR gene requires AP-1 and GRAS elements [13,20]. However, sequence homologous to the GRAS element has not been identified within the porcine promoter. Despite the presence of multiple putative AP-1 elements, mutation of these binding sites within luciferase reporter constructs and subsequent transient transfections of αT3-1 cells failed to reduce activity of the porcine GnRHR promoter (data not shown).

Another interesting aspect of porcine GnRHR regulation in gonadotrope cells is the length of promoter required for maximal basal expression. Only 500 bp of the mouse proximal promoter are required for gonadotrope-specific expression of the GnRHR [13]. In the human, expression in the pituitary appears to be conferred within 577 bp of promoter, with a placental-specific promoter being located between -1737/-1346, and a granulosa/luteal cell-specific promoter at -1300/-1018 relative to the translational start site [24,40,41]. We have shown that the proximal GSE located within 200 bp of 5′ flanking sequence is critical to expression of the porcine GnRHR gene. Yet, the -524pGL3 reporter construct was not sufficient to confer basal activity of the porcine GnRHR promoter in αT3-1 cells. However, maximal basal expression of the porcine GnRHR gene requires 1779 bp of 5′ flanking region. This indicates that spatially, the pig promoter appears to be more similar to the human than the mouse. The primary difference being that the porcine gonadotrope-specific promoter encompasses a region that in the human confers expression in three different cell types, pituitary, placental and granulosa/luteal. Typically, elements located at -1800 bp relative to the translational start site may be considered enhancers; however, the porcine GnRHR promoter elements necessary for gonadotrope-specific expression are spaced much farther apart than in the mouse and human previously described GnRHR promoters [11,23].

Retinoid receptors are part of a nuclear receptor family that can be divided into 2 subfamilies, RXR and retinoic acid receptors (RAR). While several studies have implicated RXR in the transcriptional regulation of the GnRH gene within GT1-1 neuronal cells [42]*,* there are no reports indicating RXR involvement in GnRHR gene expression*.* Further, evaluation of the human GnRH II gene promoter in neuronal (TE671) and placental (JEG-3) cell lines demonstrated that the NF-κB subunit, p65, as well as RAR/RXR dimers bind a 6-bp repressor element, designated GII-Sil [43]. Evidence that the complex nuclear receptor, RXR, alters porcine GnRHR gene expression is unique and merits further investigation.

From EMSA experiments, we determined that the upstream enhancing region is comprised of a GSE and additional elements necessary for activity of the porcine GnRHR promoter in αT3-1 cells. Sequence analysis of this region identified several putative binding sites including NF-κB and OCT1. NF-κB can interact with SF1 to inhibit Mullerian inhibiting substance gene expression [44]. The Kaiser laboratory has previously shown that OCT1 binds to the SURG-1 element within the murine GnRHR promoter for basal as well as GnRH stimulated activity [18]. In contrast, OCT1 can act as a transcriptional repressor of the human GnRHR gene [25]. Although EMSA experiments performed in our laboratory indicated that NF-κB and OCT1 were plausible transcription factors, each binding to recognition sites within the upstream enhancing region, our reporter assays were unable to verify functionality of the putative NF-κB and OCT1 binding sites in basal, gonadotrope-specific expression of the porcine GnRHR gene.

## Conclusions

**Figure 8:**
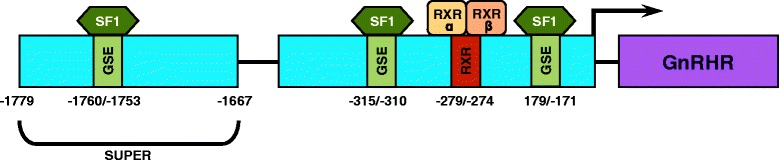
Working model for gonadotrope-specific activity of the porcine GnRHR gene promoter. In addition to the two proximal GSEs and RXR binding site that comprise the gonadotrope-specific promoter, another SF1 binding site located within the distal enhancing region of the promoter is necessary for maximal basal expression of the porcine GnRHR gene.

Activity of the porcine GnRHR promoter in αT3-1 cells is partially conferred by a distal GSE, two proximal GSEs and an RXR binding site (Figure 8). Transcriptional regulation of the porcine GnRHR gene is unique from other species in that there are three GSEs involved in basal gonadotrope expression and RXR has not previously been identified as a regulator of GnRHR promoter activity*.*

## Abbreviations

AP1: Activator protein 1
EMSA: Electrophoretic mobility shift assay
FSH: Follicle stimulating hormone
GnRH: Gonadotropin-releasing hormone
GnRHR: Gonadotropin-releasing hormone receptor
GnSE: GnRHR specific enhancer
GRAS: GnRH receptor activating sequence
GSE: Gonadotrope specific element
LH: Luteinizing hormone
LHX3: LIM homeobox 3
NF-κB: Nuclear factor-κB
NF-Y: Nuclear factor Y
OCT1: Octamer transcription factor 1
PCR: Polymerase chain reaction
Pitx-1: Pituitary homeobox 1
RAR: Retinoic acid receptor
RSV: Rous sarcoma virus
RXR: Retinoid X receptor
SAP: SF1 adjacent protein
SF1: Steroidogenic factor 1
StAR: Steroidogenic acute regulatory protein
SURG-1: Sequence underlying responsiveness to GnRH
UTR: Untranslated region
